# Psychological Characteristics of Children of Alcoholics

**Published:** 1997

**Authors:** Kenneth J. Sher

**Affiliations:** Kenneth J. Sher, Ph.D., is Frederick A. Middlebush Professor of Psychology at the University of Missouri, Columbia, Missouri

**Keywords:** children of alcoholics, psychological development, behavioral and mental disorder, AODU (alcohol and other drug use) development, family AODU history, childhood, adulthood, fetal alcohol syndrome, research, personality, risk factors, literature review

## Abstract

More than 20 years ago, researchers first noted that children of alcoholics (COA’s) appeared to be affected by a variety of problems over the course of their life span. Such problems include fetal alcohol syndrome, which is first manifested in infancy; emotional problems and hyperactivity in childhood; emotional problems and conduct problems in adolescence; and the development of alcoholism in adulthood. Although much has been learned over the ensuing two decades, a number of controversial research areas remain. In particular, debate stems from the fact that despite a common interest in COA’s, clinically focused literature and research-focused literature have resulted in two distinct bodies of knowledge. This article reviews important research results, with emphasis on findings generated by the alcohol-research community. Attention also is given to examining the empirical validity of concepts that have been advanced by several influential clinicians from the COA field.

At least two important constituencies have generated interest in the psychological characteristics of children of alcoholics^1^ (COA’s). One is the community of clinicians, consisting of mental health and addiction workers and, to some extent, the general public. A number of influential clinicians (see, for example, Black 1982) have described COA’s as victims of an alcoholic family environment characterized by disruption, deviant parental role models, inadequate parenting, and disturbed parent-child relationships. These family related variables are thought to undermine normal psychological development and to cause distress and impaired interpersonal functioning, both acutely and chronically. Most of the descriptions of COA’s, however, have been based primarily on anecdotal reports of people seeking help for any number of psychological or behavioral problems.

A second constituency studying COA’s is the research community, which is seeking to understand the causes of alcoholism. COA’s are at substantially increased risk for becoming alcoholic themselves, and this elevated risk appears to be a function of both genetic and environmental factors (Heath 1995; see also the article by McGue, pp. 210–217). By identifying characteristics that distinguish COA’s from children of non-alcoholics (non-COA’s), researchers hope to identify variables that might be important in the etiology of alcoholism. Most of these descriptions are based on data obtained relatively systematically from nonclinical and clinical populations.

Despite a common interest in COA’s, the literature based on clinicians’ experiences and the literature from the community of researchers have not overlapped to any great extent and have provided two distinct bodies of knowledge. This article primarily focuses on findings generated by the alcohol-research community. In addition, because of the effect that some of the clinical writings have had on both the community of practitioners and the lay public, this article also examines the empirical validity of some of the concepts put forth by influential clinicians.

## Factors Influencing COA Research

Although researchers have examined the possible relationship between family history of alcoholism and its effects on the adaptation of offspring since the beginning of this century (MacNicholl 1905), widespread interest in the problems of COA’s did not appear to gain much momentum until the 1960’s. By the mid-1970’s, however, a sufficiently large number of empirical findings permitted El-Guebaly and Offord (1977) to document a wide range of problems encountered by COA’s across the life span, including fetal alcohol syndrome (FAS),^2^ which is first manifested in infancy; emotional problems and hyperactivity in childhood; emotional and conduct problems in adolescence; and alcoholism in adulthood. In the past 20 years, research has advanced on several fronts and has helped to clarify the nature and extent of problems facing COA’s as well as the numerous variables that must be considered when attempting to make generalizations about this group (Sher 1991; Windle and Searles 1990).

In fact, perhaps the most significant revelation about COA’s that the research community has established is how difficult it is to make valid generalizations. A number of reasons exist for this situation. Most significantly, alcoholics do not represent a homogeneous class of people. Many other psychological disorders coexist (i.e., are comorbid) with alcoholism. These disorders include other forms of substance use disorders (i.e., drug use disorders), anxiety disorders, mood disorders, and personality disorders (National Institute on Alcohol Abuse and Alcoholism [NIAAA] 1993). Thus, some COA’s also are children of depressives, children of agoraphobics, children of people with antisocial personality disorder, and so forth.

Given the many forms of psychopathology that are possible in parents of COA’s, difficulties often arise in attributing any apparent COA characteristic specifically to parental alcoholism. This general principle was illustrated in a classic family history study that Winokur and colleagues conducted nearly 30 years ago (Winokur et al. 1971). They examined the prevalence of alcoholism, depression, and sociopathy^3^ in the mothers, fathers, and siblings (i.e., first-degree relatives) of (1) alcoholics whose comorbid psychological problems, if any, developed after their alcoholism (i.e., they were “primary” alcoholics); (2) alcoholics with primary depression; and (3) alcoholics with primary sociopathy. The researchers’ findings revealed that “in first-degree family members, alcoholism [was] more frequently seen for the primary alcoholism group, depression for the depression alcoholism group and sociopathy for the sociopathy alcoholism group” (p. 531). In other words, the alcoholic’s comorbid psychopathology was critical in predicting the psychopathological outcomes in relatives.

Moreover, even in the absence of significant comorbidity, considerable differences (i.e., heterogeneity) exist among alcoholics. Researchers have proposed numerous approaches to conceptualizing heterogeneity among alcoholics, incorporating a range of dimensions such as age of onset, drinking pattern, extent of antisociality, severity of dependence, personality traits, and even family history (Babor et al. 1994). Although no consensus exists on the optimal classification of alcoholics or even on whether such heterogeneity is best conceptualized as a number of distinct “subtypes” or as a number of interacting dimensions, researchers generally agree that alcoholics vary widely along almost any clinically relevant variable. As demonstrated by Winokur and colleagues (1971), parental characteristics above and beyond alcoholism are important determinants of features observed in the alcoholics’ offspring.

Although heterogeneity of parental alcoholism is one key class of variables that must be recognized, numerous other domains need to be considered when evaluating the relation of parental alcoholism to offspring outcomes. First, considerable variability exists in the characteristics of siblings from any family. That is, two children can share the same biological parents and general rearing conditions yet be profoundly different along multiple psychological dimensions, even on characteristics known to be moderately heritable. Thus, even if relatively homogeneous classes of alcoholics (and their spouses) could be identified, considerable variability would be expected in their offsprings’ characteristics.

Second, the extent to which parental alcoholism is associated with specific characteristics in the offspring largely depends on the group with which COA’s are being compared. Although it is reasonable to simply ask whether COA’s differ from non-COA’s, the answer to this question does not reveal whether COA’s are unique compared with the children of parents with other major psychological or behavioral problems. Indeed, accumulating evidence reveals that children from families with a range of problems show a number of similar deficits.

Additionally, many methodological complexities exist, including the way in which alcoholism is measured in the parent(s), how extensively alcoholism is assessed in other family members, whether parental alcoholism is “active” or in recovery, the way in which subjects are sampled and ascertained, the sex of the alcoholic parent, and the age and sex of the child. This short list is by no means exhaustive. The implications of many of these methodological issues are discussed at length by Sher (1991). These complexities make it difficult to draw strong generalizations concerning the psychological characteristics of COA’s. It is, therefore, not surprising that the research literature is marked by a number of contradictory findings. Nevertheless, careful scrutiny of the literature reveals sufficient consistency in certain areas to offer some supportable generalizations. One must keep in mind, however, that such generalizations apply only to heterogeneous groups of people identified as COA’s, that more homogeneous classes of COA’s might not fit the generalization well, and that any individual COA might not fit the generalization at all. With these caveats in mind, this article reviews two important classes of psychological variables—psychopathology and personality—that have been extensively investigated in recent years.

## Psychopathology in COA’s

As mentioned earlier, more than 20 years ago, El-Guebaly and Offord (1977) noted that COA’s appeared to be affected by a variety of problems throughout their lives. Although much has been learned over the ensuing two decades, several areas of controversy remain.

### Internalizing and Externalizing Symptoms

Researchers have identified two broad classes of psychopathological symptoms in childhood: internalizing and externalizing symptoms. Internalizing psychopathology encompasses symptoms such as anxiety and depression. A number of studies show that COA’s report high levels of depression and anxiety. As noted by West and Prinz (1987), however, it is unclear whether these adjustment problems are directly related to a parent’s alcoholism, indirectly related by way of family disruption, or spuriously related (e.g., resulting from parental comorbidity or common genetic makeup [i.e., genotype]) (see Kendler et al. 1995).

Externalizing psychopathology primarily encompasses “acting out” types of behavior—characterized by rule breaking, defiance, aggression, inattention, and impulsivity—and corresponds to what is termed “attention deficit and disruptive behavior disorders” (i.e., attention deficit/hyperactivity disorder [ADHD], oppositional defiant disorder [ODD], and conduct disorder [CD]) in the DSM–IV. Unfortunately, much of the early research on family history of alcoholism and these behavior disorders was conducted before many of today’s accepted diagnostic distinctions were made. Consequently, the literature is considerably less precise than is desirable.

High comorbidity between ADHD and CD makes it difficult to specify the exact nature of externalizing psychopathology among COA’s. Nevertheless, COA’s, as a group, show many characteristics associated with this broad class of disorders (Pelham and Lang 1993; Pihl et al. 1990; Sher 1991; Windle 1990). This finding is not surprising, given that externalizing psychopathology is associated with a range of parental diagnoses (especially parental antisocial personality disorder) and with a range of family stressors. What is most uncertain is the exact nature of the association. Frick (1994) attempted to identify specific family variables that appear to be uniquely associated with different forms of externalizing psychopathology. He concluded that both parental substance use and antisocial behavior are associated with CD in offspring. When studies statistically controlled for CD, however, no relation was found between ADHD and parental antisociality and substance use.^4^ In general, Frick also did not find any family variables that distinguished between ODD and CD. Thus, it appears that CD is important in the intergenerational transmission of alcohol use disorders. ADHD is important only to the extent that it is a risk factor for CD.

Many potential explanations can be found for the association between parental alcoholism and children’s externalizing disorders. Such explanations include the effects of alcohol on family disruption and parenting, comorbidity of parental alcoholism with antisocial personality disorder, a common genetic predisposition, and even reverse causation (i.e., externalizing behavior problems in a child contribute to a caregiver’s alcohol use) (Pelham and Lang 1993).

Despite provocative findings concerning the link between parental alcoholism and childhood behavior problems, the existing database is limited. Relatively few studies have included careful diagnoses of large samples of parents and children, and these samples rarely are followed over time. However, this situation is changing rapidly. Within the next few years, researchers should be able to make more specific and definitive conclusions.

## Psychopathology in Adulthood

Despite the inconclusive and controversial findings on COA’s cited earlier, scientists and others do agree that COA’s themselves are at high risk for developing alcoholism (Heath 1995; McGue 1993). In fact, there is probably no more robust finding in the area of alcohol research than the finding that COA’s are somewhere between 2 and 10 times more likely to develop alcoholism than non-COA’s. Alcoholic fathers tend to increase the risk for alcoholism in both their sons and their daughters, whereas the risk associated with maternal alcoholism seems to be more limited to daughters (Pollock et al. 1987). Additionally, parental alcoholism appears to be associated with other substance use disorders in their offspring, such as drug abuse, drug dependence, and tobacco dependence, although the evidence is not as extensive (see Gotham and Sher 1996*a*).

Findings regarding the extent to which COA’s report high levels of anxiety and depression are more controversial. For example, two major reviews (Kushner et al. 1990; Schuckit and Hesselbrock 1994) in the same journal arrived at conflicting views concerning whether COA’s are at risk for anxiety disorders. Likewise, in a recent issue of *Alcohol Health & Research World*, Schuckit (1996) and Merikangas and colleagues (1996) derived opposite conclusions concerning whether the relatives of alcoholics are at elevated risk for anxiety disorders. This disagreement stems from many sources, including variability in the sources of samples studied; the extent to which anxiety symptoms represent short-term consequences of alcohol consumption, alcohol withdrawal, or an underlying disorder; and the influence of choosing spouses or partners who are prone to anxiety states. At this point, it is probably fair to conclude that although much data suggest that COA’s are at elevated risk for both depression and anxiety, a number of unanswered questions remain. Researchers clearly need more data before they can make definitive conclusions.

Another significant but little-researched area concerns the extent to which COA’s are at increased risk for personality disorders. Personality disorders are characterized by disturbed interpersonal relationships that result in difficulties such as trouble with the law, family conflict and discord, and impairment in both social and occupational functioning. The dearth of data on personality disorders among COA’s constitutes a significant gap in the scientific literature. The topic is especially relevant, however, because both clinical literature and lay descriptions often focus on constructs such as codependency (see the section “Personality Characteristics of COA’s: The Clinical Literature”).

Although COA’s appear to be at high risk for the development of antisocial personality disorder, findings are unclear as to whether this increased risk is attributable to the fact that the COA’s studied were offspring of alcoholics with comorbid antisociality (Sher 1991). Whether COA’s are at high risk for other personality disorders also is unclear. Drake and Vaillant (1988) compared COA’s and non-COA’s on the overall rate of personality disorder as well as on rates of individual personality disorders and failed to find differences. However, this study was limited by the exclusionary criteria applied at the beginning of the study (i.e., neither men nor women with conduct disorder were followed) and by the narrow age range of the subjects at assessment (the participants’ average age was 47). Moreover, several studies have demonstrated that approximately 40 percent of borderline personality disorder patients have at least one alcoholic first-degree relative (see, for example, Cowdry and Gardner 1988) or that the morbid risk for alcoholism among first-degree relatives of borderline patients is substantial (see, for example, Links et al. 1988). Thus, with the notable exception of antisocial personality disorder, research on the relationship between a family history of alcoholism and personality disorder is sparse. Existing data suggest, however, that further inquiry in this area is needed.

## Personality Characteristics of COA’s: The Research Literature

The personality characteristics of COA’s have been a focus of the alcohol research community because influential theorists (see, for example, Cloninger 1987) have speculated that much of the heritability for alcoholism is mediated by personality traits. In other words, COA’s might be expected to differ from non-COA’s on key personality dimensions, differences that might explain the COAs’ risk for alcoholism and other behavioral problems.

Although personality-based explanations of psychopathology have existed since ancient times, the emphasis on behavioral models among psychologists and the focus on medical or biological models among psychiatric researchers marginalized these explanations for a number of years (Maher and Maher 1994). A resurgence of interest currently exists, however, in the relationship between personality and psychopathology: Many theorists now view personality as a key to understanding a range of common psychological disorders (Watson and Clark 1994). This view is compatible with both biological and behavioral perspectives. For purposes of this discussion, the general term “personality” is used to describe a large class of variables that includes both very basic, highly heritable traits evident early in development (i.e., temperament) and traits more commonly associated with variation in adult personality. Although considerable variability exists in how different theorists define personality, most formal definitions note that personality is “internal, organized, and characteristic of an individual over time and situations … [and has] motivational and adaptive significance” (Watson et al. 1994, p. 18).

Over the past two decades, scores of studies, using hundreds of specific (and often narrow) personality measures, have been published on the personality characteristics of COA’s. To provide an overview, this article addresses three broad categories of personality traits: neuroticism/negative emotionality, impulsivity/disinhibition, and extraversion/sociability. Although debate continues on the optimal number of categories required to adequately reproduce the dimensions of personality descriptions encompassed by everyday language, the three categories have considerable empirical support (Zuckerman et al. 1988). Moreover, they can be subsumed and readily translated into four- (Watson et al. 1994) and five- (Digman 1990) factor models of personality that are becoming increasingly accepted by the scientific community.

### Neuroticism/Negative Emotionality

This category includes personality traits such as a tendency to experience negative affective states (e.g., depression and anxiety), a propensity for guilt and self-blame, and sensitivity to criticism. Cross-sectional studies of COA’s reveal mixed support for differences on this personality dimension. For example, studies using Eysenck’s Neuroticism scale yield contradictory findings. Some studies have found that COA’s are more neurotic than non-COA’s; other studies show no differences between the two groups. More generally, COA’s at high risk for alcoholism have not been found to report high levels of anxiety (Sher 1991). Although COA’s often report relatively high levels of depression, this state appears to be situational and tied to the active drinking of an alcoholic parent (Moos and Billings 1982).

Related to the dimension of neuroticism/negative emotionality is the variable of self-esteem. In general, COA’s appear to have lower self-esteem than non-COA’s in childhood, adolescence, and young adulthood (Sher 1991). Self-esteem deficits could reflect high levels of neuroticism or possibly transient depressive states. Thus, although evidence exists that COA’s may be characterized by higher levels of negative emotionality than non-COA’s, the majority of negative findings in this area cast doubt on the robustness of neuroticism as a reliable discriminator of heterogeneous samples of COA’s versus non-COA’s (Windle 1990). Parental comorbidity, however, may exercise a significant influence on the magnitude of differences between COA’s and non-COA’s, with neuroticism in offspring most commonly associated with alcoholic parents who have comorbid anxiety and depressive conditions. Moreover, active alcoholism in a parent can lead to family disruption and result in transient increases in anxiety and depression. These reactive mood states, however, should not be interpreted as necessarily indicating more enduring trait characteristics.

### Impulsivity/Disinhibition

The personality category that appears to be most associated with being a COA is that of impulsivity/disinhibition, which encompasses traits such as sensation seeking, aggressiveness, and impulsivity. Numerous cross-sectional studies (see, for example, Pihl et al. 1990; Sher 1991; Windle 1990) indicate that antisocial, aggressive, and impulsive traits characterize the offspring of alcoholics. These same traits also appear to be those that are most associated with the development of alcoholism, suggesting that these personality characteristics might represent important mediators of the intergenerational transmission of alcoholism (e.g., Sher and Trull 1994).

Interpretation of these findings is not unambiguous, however. For example, Nathan (1988) has questioned the motivational basis of these characteristics and has concluded that “it is primarily behavior and not personality” (p. 187) that is reflected by these measures. Although Nathan’s concern with the personality-based interpretation of many behavioral indicators of impulsivity/disinhibition is appropriate, it may be overstated. An increasing number of studies demonstrate that differences between COA’s and non-COA’s on personality questionnaire measures of impulsivity/disinhibition do not directly ask about deviant behaviors (Sher et al. 1995). At present it seems reasonable to conclude that traits related to impulsivity/disinhibition are important correlates of being a COA. In most studies, however, the magnitude of this association is not great, and it is possible that much of the association is attributable to comorbid antisociality tendencies in the alcoholic parent.

### Extraversion/Sociability

The dimension of extraversion/sociability (also sometimes referred to as positive emotionality or positive affectivity) encompasses traits such as gregariousness, sociability, dominance, and energy. This characteristic has not been found to reliably distinguish COA’s from non-COA’s (Sher 1991; Windle 1990), a finding that is somewhat surprising because extraverted traits have been shown to predict both frequent intoxication and later drinking problems. Data suggest that as people become increasingly alcohol dependent, they become more introverted (Sher and Trull 1994). Consequently, it is possible that the failure to find reliable differences between COA’s and non-COA’s on extraversion/sociability stems, in part, from failure to control for alcohol dependence that could mask this trait. Alternatively, the seeming sociability of some prealcoholics might be more a reflection of disinhibition rather than true sociability (Tarter 1988).

A number of other personality traits (e.g., difficulty in recognizing and describing emotions [alexithymia], locus of control,^5^ type A personality, and self-consciousness) do not fit neatly into any of the three broad categories noted above and have, at least in one study, been found to distinguish COA’s from non-COA’s (Sher 1991; Windle 1990). Currently, however, inconsistent findings and a relatively small database make it difficult to draw any strong conclusions in this area.

Thus, the research literature, although far from conclusive, suggests that traits associated with impulsivity and disinhibition are related to a family history of alcoholism. The magnitude of this effect is not large, however, and these traits might be primarily associated with only certain forms of parental alcoholism. These personality findings are consistent with the developmental psychopathology literature, which indicates that COA’s are at an excess risk for disruptive behavior problems, especially conduct disorder. It also is important to note that personality traits can be linked to various etiological processes (for an extended discussion, see Sher 1991). For example, consider that impulsivity/disinhibition could lead to impaired control over alcohol consumption, greater sensitivity to alcohol’s effects, and general nonconformity. Neuroticism/negative emotionality is associated with affective disturbance (i.e., both depression and anxiety), which could lead people to self-medicate their distress with alcohol. Extraversion/sociability may be associated with greater involvement in social activities where alcohol is consumed and thus increase a person’s exposure to heavy drinking within social contexts. This list, though by no means exhaustive, gives an indication of the wide range of effects associated with various personality traits.

## Personality Characteristics of COA’s: The Clinical Literature

Clinicians have described a number of personality variables purported to characterize COA’s and to result in long-term adjustment difficulties. Many personality descriptors have been applied to COA’s, especially to adult COA’s (or ACOA’s). These descriptors appear to be embraced by many clinicians as well as by numerous people who have grown up with alcoholic parents. As previously noted, however, the research literature does not indicate that COA’s as a group show significant personality deviance. Although findings indicate that some COA’s may be characterized by disinhibition/impulsivity (and possibly neuroticism/negative emotionality), the magnitude and consistency of these observed differences are not consistent with the portrayals presented in some clinical texts (Brown 1988) and trade publications (Cermak 1988; Woititz 1984).

How then can we explain this seeming discrepancy? More than 40 years ago, Paul Meehl (1956) noted that people tended to accept a personality description as valid merely because it was so vague, double-headed, socially desirable, or widely occurring in the general population that it defied rejection. This type of personality description, although likely to receive high rates of acceptance, is also likely to be of little clinical value because it lacks the descriptive specificity and prognostic utility necessary to differentiate people. Meehl (1956) termed these types of descriptions “Barnum” statements, in honor of the noted showman P.T. Barnum’s recipe for putting on a successful circus—make sure there’s a little something in it for everybody. In fact, many of the COA descriptors presented in the literature appear to possess the features of classic Barnum statements. For example, the descriptors might be vague (e.g., COA’s have difficulty determining what normal is) (Woititz 1984) or double-headed (e.g., COA’s are either super responsible or super irresponsible) (Woititz 1984); express socially desirable attributes (e.g., COA’s are sensitive to the needs of others) (Black 1982); or describe attributes that occur with high frequency in the general population (e.g., COA’s are uncomfortable when they are the center of attention) (Black 1982).

To test the hypothesis that popular ACOA descriptors function like classic Barnum statements, Logue and colleagues (1992) had groups of young adult COA’s and non-COA’s complete computer-administered personality inventories. The groups then were presented with personality profiles purportedly generated automatically based on their responses. These profiles, however, actually consisted of descriptions based on generalizations drawn from either the clinical ACOA literature or from statements used in the Barnum literature. A key finding was that all subjects rated the ACOA profiles as highly descriptive of themselves regardless of their family history. Second, little difference existed in the self-descriptiveness ratings of ACOA and Barnum profiles, further suggesting that the ACOA descriptors appeared to function as Barnumlike statements. Third, and consistent with the larger literature on the Barnum effect, the personality descriptions were seen as better descriptions of the self than of people in general. That is, participants found these COA descriptions to be somewhat specific descriptions of themselves. This study illustrates why it should not be too surprising that many COA’s (and ACOA’s) find the portrayals in the media to be accurate descriptions of themselves. These characteristics are viewed as descriptive by most people, COA and non-COA alike. It is vital, then, not to confuse this perceived descriptiveness with scientifically valid descriptions.

Perhaps the most popular concept to emerge from the COA literature is that of codependency. Gordon and Barrett (1993) note that codependency was first described as a “disease” of “compulsive caretaking” found in spouses of alcoholics. The meaning of the term has now been broadened to include COA’s and nearly anyone involved in a relationship with an alcoholic or with someone with significant problems (e.g., psychopathology or illness). Although a number of alternative conceptualizations of codependency can be found, Wright and Wright (1991) note that the most popular notion is as a personality syndrome composed of denial, constriction of emotions, depression, hypervigilance, compulsions, and a number of other characteristics.

What evidence is there to support the validity of the concept of codependency? Despite the recommendation of an Institute of Medicine (1989) report that “researchers should be encouraged to investigate popular trends and concepts in the treatment field—for example, the need for special treatment services for adult children of alcoholics and codependents” (p. 146)—relatively minimal scientific study has been conducted on codependency. Moreover, studies that have addressed the topic tend to have serious methodological limitations (e.g., especially in the area of sampling), examine only a narrow aspect of purported codependent characteristics, and fail to demonstrate whether the concept of codependency has additional explanatory value beyond the established constructs in the area of psychopathology (and, thus, have not demonstrated the usefulness of the concept).

In one recent study, Gotham and Sher (1996*b*) administered a self-report inventory of codependent traits, along with measures of personality and psychopathology, to a large sample of young adult COA’s and non-COA’s. Although the codependency measure was significantly (but not strongly) related to having an alcoholic father, most of the association appeared to be attributable to a general factor of neuroticism/negative emotionality. Indeed, the codependency scale was strongly correlated with a frequently used measure of neuroticism. However, even after statistically controlling for basic dimensions of personality and psychopathology, a small but significant association between family history and codependent traits remained. These traits included denial and feelings of having been cheated or “let down.” These findings were not unexpected, because many COA’s experience anger and resentment as a result of family disruption. Additional research is needed to determine whether the concept of codependency describes a cluster of traits unique to COA’s or represents more general issues of growing up in a disrupted home (Gotham and Sher 1996*b*).

## Conclusion

To date, existing research indicates that care should be taken when making generalizations about the psychological characteristics of COA’s. Clearly, evidence indicates that as a group, COA’s are at higher risk than non-COA’s for a number of psychological disorders in both childhood and adulthood and that they seem to be more impulsive and possibly more neurotic than people without alcoholic parents. With the exception of the risk for substance use disorders, however, the proportion of COA’s affected by these other psychological disorders does not appear to be large. Furthermore, it is potentially harmful (Burk and Sher 1988) to infer much about a specific person based solely on his or her family history of alcoholism. Thus, many of the popular portrayals of COA’s are clearly overgeneralizations and have the potential to be harmful.

The more that is known about other elements of a person’s family history (e.g., the number of family members who are alcoholic or who have disorders frequently comorbid with alcoholism) and, more important, about the details of the person’s behavior, the more valid the statements will be about his or her personality and psychological adjustment. From this perspective, simply knowing that someone is a COA represents no more than a starting point for obtaining more in-depth information.

## Footnotes

1 Definitions of alcoholism can vary in the alcoholism literature. Generally, a person clinically diagnosed with alcohol abuse or alcohol dependence must meet specific criteria (e.g., as defined by the American Psychiatric Association’s [1994] *Diagnostic and Statistical Manual of Mental Disorders, Fourth Edition* [DSM–IV]). The term “alcoholism” in this article, however, is used to encompass all levels of problem alcohol use and does not refer to a particular diagnostic system.

2 An FAS diagnosis is made when an alcohol-exposed infant exhibits pre- or postnatal growth deficiency, a specific pattern of anomalies in the structure of the face and skull, and some central nervous system dysfunction (Mattson and Riley 1995).

3 Sociopathy is a diagnostic category similar to the category of antisocial personality disorder. Antisocial personality disorder is a pattern of irresponsible and antisocial behavior that begins early in life and continues through adulthood.

4 The lack of a relationship between these variables does not mean that COA’s do not have higher levels of ADHD than control subjects, only that such an association can be “explained” by the parental conduct disorder.

5 The concept of locus of control refers to the tendency to attribute control over one’s life either to oneself or to external factors.
